# First record of the genus *Eulocastra* Butler, 1886 (Lepidoptera, Noctuidae) from the Indochina Peninsula, with notes on the distribution and host associations of *Eulocastra sasakii* Sugi, 1985

**DOI:** 10.3897/BDJ.14.e214068

**Published:** 2026-10-26

**Authors:** Yeong-Bin Cha

**Affiliations:** 1 Forest Biodiversity Research Division, Korea National Arboretum, Pocheon, Republic of Korea Forest Biodiversity Research Division, Korea National Arboretum Pocheon Republic of Korea

**Keywords:** endangered species, endemic species, Eustrotiinae, host plants, IUCN, Oriental Region

## Abstract

**Background:**

*Eulocastra sasakii* Sugi, 1985 (Lepidoptera, Noctuidae) was previously known only from Japan and has, therefore, been regarded as a Japanese endemic species. The species is considered threatened in Japan and is currently listed as Vulnerable (Category II) at the national level and in regional Red Data Books. Although both male and female specimens were included in the original description, only the male genitalia were described and illustrated, leaving the female genitalia unknown. Furthermore, *Molinia japonica* Hack., a temperate East Asian grass, has been reported as the only known host plant of the species.

**New information:**

The genus *Eulocastra* Butler, 1886 and *E. sasakii* Sugi, 1985 are recorded from Cambodia and the Indochina Peninsula for the first time. Adults of both sexes, the wing venation and the male genitalia are illustrated and the female genitalia are described and illustrated for the first time. This record extends the known distribution of the species from Japan to Southeast Asia and raises the possibility of an alternative host plant association in Cambodia.

## Introduction

The genus *Eulocastra* Butler was established, based on the Australian species, *E. fasciata* Butler by monotypy. Most species of the genus are distributed from Africa to Eurasia, except *E. chrysarginea* (Schaus) and *E. ecphaea* Dognin. In East Asia, four species have been recorded: *E. excisa* (Swinhoe), *E. latifasciata* Wileman, *E. sasakii* Sugi and *E. shibaharai* Kishida (Kishida 2011). Of these, three species, excluding *E. excisa*, have been recorded only from Japan. Notably, *E. latifasciata*, described from Japan in 1911, remains particularly poorly known and has not been recorded since its original description (Sugi 1990, Kishida 2011). Solis (1992): 283 subsequently treated this species in the genus *Lepidogma* (Pyralidae, Epipaschiinae), but provided no discussion supporting this placement. Following the more recent treatment of Kishida (2011), we here retain *E. latifasciata* in *Eulocastra*.

*Eulocastra sasakii* Sugi, 1985 was first reported from Akita Prefecture, Japan, as *Eublemma* sp., based on a single female specimen (Sugi 1982) and was subsequently described as a new species by Sugi (1985), based on this female and an additional male specimen. Although female specimens were available to Sugi, only the male genitalia were illustrated and described and the female genitalia have remained unknown (Sugi 1982, Sugi 1985). Sugi (1990) subsequently noted the external similarity of *E. sasakii* to *E. quintana* (Swinhoe), a species described from India. However, the type specimen of *E. quintana* has not been located and the species remains poorly known, with no subsequent specimens or detailed morphological information available for comparison beyond the original description and illustration.

Within Japan, *E. sasakii* is regarded as a rare species and is included in several national and prefectural Red Data assessments, Vulnerable (Category II) by the Ministry of the Environment of Japan; Red Data (NT) in Aichi, Mie and Gifu Pref.; Red Data (EN) in Akita Pref.; and notable species in Fukui Pref. (Fukui Prefecture’s own classification system) (Gifu Prefecture 2010, Fukui Prefecture 2016, Aichi Environmental Research Center 2020, Akita Prefecture Department of Living Environment. Nature Conservation Division 2020, Ministry of the Environment, Japan 2020, Mie Prefecture 2025).

During examination of noctuid material collected in Cambodia, three specimens, comprising two males and one female, were found to agree closely with *E. sasakii* in external morphology. Examination of the genitalia further supported their identification as *E. sasakii*. The female specimen also provided an opportunity to document the previously unknown female genitalia of the species.

Consequently, we report the genus *Eulocastra* Butler and *E. sasakii* Sugi from Cambodia and the Indochina Peninsula for the first time, representing the first record of the species outside Japan. We provide re-descriptions and illustrations of adults and genitalia of both sexes, including the first description and illustration of the female genitalia and discuss the diagnostic characters and distribution of the species.

## Materials and methods

All voucher specimens were deposited in the private insect collection of the author.

Images of adults were taken by a Tucsen Dhyana 400DC digital camera (Fuzhou, China) attached to a Leica S6D stereomicroscope with dome illuminator Leica LED5000 HDI (Wetzlar, Germany). Genitalia were dissected and examined under the Leica EZ4 stereomicroscope and Leica Investa3 stereomicroscope. Genitalia photographs were taken using a Tucsen Dhyana 400DC digital camera mounted on a Leica S8APO stereomicroscope with Mosaic software (version 2.3, Tucsen).

Attempts were made to extract DNA from the examined specimens; however, no usable sequences were obtained, likely due to specimen age (collected approximately 15 years ago).

### Institutional abbreviation

NHMUK = Natural History Museum, London, United Kingdom

## Taxon treatments

### Eulocastra sasakii

Sugi, 1985

C4C76C5A-EE79-5A6A-9FE8-F19E10C92AF2

Eulocastra sasakii Sugi, 1985 - *Sugi (1985)* : 90. Holotype: male, in coll. NHMUK.
Eublemma sp.: Sugi (1982): 809.
Eulocastra sasakii Sugi: Sugi (1990): 99; Nakamura et al. (1996): 228; Gifu Prefecture (2010): 0005227; Kishida (2011): 206; Fukui Prefecture (2016): 224; Aichi Environmental Research Center (2020): 425; Akita Prefecture Department of Living Environment. Nature Conservation Division (2020): 70; Ministry of the Environment, Japan (2020): 25; Mie Prefecture (2025): 286.

#### Materials

- **Type status:** Other material. **Occurrence:** catalogNumber: INU-12214, CYBG-0244; recordedBy: Y.S. Bae, Y.K. Kim, Y.D. Ju, B.S. Park, H.J. Lee; sex: 1 male, 1 female; lifeStage: adult; occurrenceID: 9047AA3C-789E-51EC-AAFB-C163CC5B673C; **Location:** higherGeography: Cambodia; Seima; **Event:** year: 2011; month: 11; day: 09; **Record Level:** type: PhysicalObject; basisOfRecord: PreservedSpecimen
- **Type status:** Other material. **Occurrence:** catalogNumber: CYBG-0245; recordedBy: (Bae et al.); sex: 1 male; lifeStage: adult; occurrenceID: 3BF14E42-D981-5597-A8AB-E5321A8DABCC; **Location:** higherGeography: Cambodia; Seima; **Event:** year: 2011; month: 6; day: 30; **Record Level:** type: PhysicalObject; basisOfRecord: PreservedSpecimen

#### Description

HABITUS (Fig. 1a, b, c) – Length of fore-wing 6–7 mm in male, 7 mm in female; wingspan 13–15 mm. Antennae filiform in both sexes. Head creamy to metallic dark brownish-grey; ocellus present; labial palpus bright ochre to dark ochre. Thorax creamy to metallic dark brownish-grey, with two very faint greyish bands. Fore-wing ground colour creamy-white; basal area dark grey; sub-basal line black, interrupted, inwardly curved; medial band dark grey, distal half paler than proximal half, with a black discal dot; postmedial line dim, not reaching the subcostal vein; three subterminal patches dark grey, large at the costa, small and indistinct at the tornus; adterminal line grey, diffused, not reaching subterminal area; terminal line black, dotted; cilia grey to dark grey. Hind-wing ground colour creamy-ivory.

Wing venation (Fig. 1d) – Fore-wing with Sc reaching costa near 3/4; R_1_ free; areole present; R_2_ arising from the upper margin of the areole; R_3_ and R_4_ stalked almost half-length; R_5_ arising from the lower margin of the areole; M_1_ arising from the upper distal angle of the cell; M_2_, M_3_ and CuA_1_ well separated, M_2_ free, arising from near M_3_; CuA_2_ arising from the lower margin of the cell near 3/4; 1A+2A reaching near tornus, 3A absent. Hind-wing with Sc+R_1_ shortly anastomosed with Rs near base; Sc+R_1_ reaching near apex; Rs and M_1_ shortly stalked; M_2_ free, arising from near M_3_; M_3_ and CuA_1_ only shortly stalked; CuA_2_ arising from the lower margin of the cell near 2/3; 1A+2A reaching near tornus, 3A absent.

Male genitalia (Fig. 2) – Uncus elongated, beak-shaped, apically pointed. Scaphium with a pair of linear sclerites; subscaphium weakly sclerotised. Tegumen moderately sclerotised. Transtilla represented by paired rod-like arms fused dorsally with the diaphragmal membrane; diaphragmal membrane weakly sclerotised, with a granulated field; juxta well-sclerotised, inverted T-shaped. Valva elongated, tongue-shaped, asymmetric, slightly expanded distally; costal margin sclerotised, wavy, with a median protrusion, bearing a cluster of setae; right valva with a well-sclerotised axe-shaped ampullar process connected to the costal protrusion; cucullus broad, blunt in the right valva and rounded in the left valva; inner surface partially membranous, broader in the right valva; distal part of valva weakly sclerotised; harpe broad and flattened, gradually tapering distally to an acute apex. Sacculus stout, weakly sclerotised. Vinculum broadly V-shaped. Aedeagus arched, distal margin partially sclerotised; with a single spear-shaped cornutus. Eighth tergite with a reversed Y-shaped sclerotised plate; eighth sternite with a rectangular marginal sclerotised plate.

**Figure 3.**
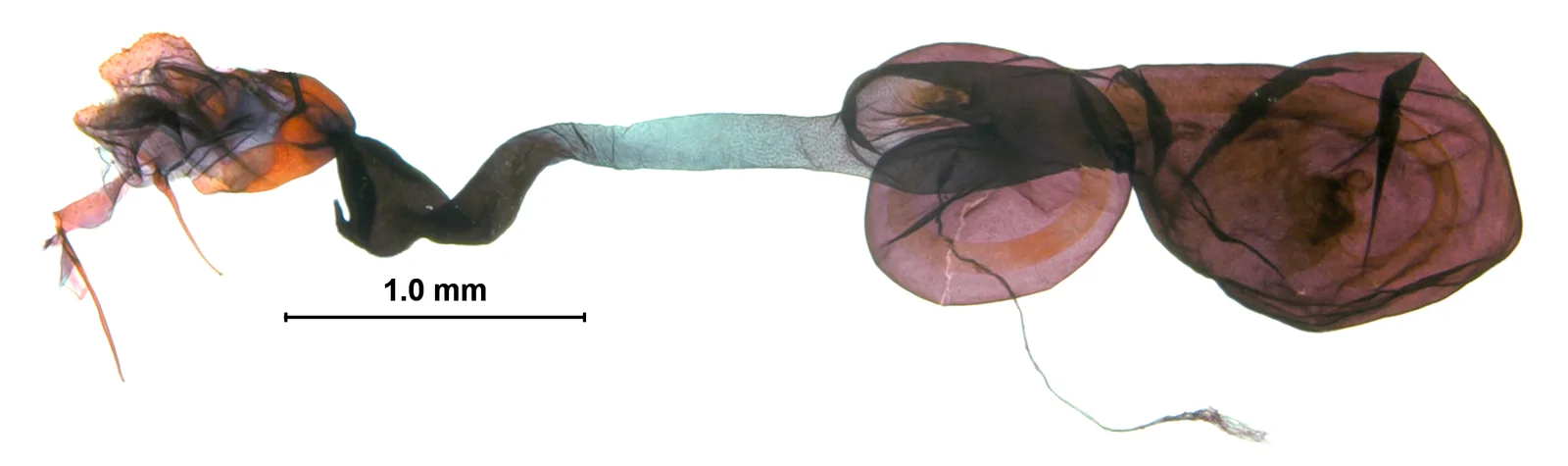
Female genitalia of Cambodian *Eulocastra sasakii* (CYBG-0244). Scale bar 1 mm.

Female genitalia (Fig. 3) – Papillae anales short. Apophyses anteriores almost as long as apophyses posteriores. Ostium bursae well-sclerotised, subtriangular, laterally folded, twisted at base, with a small asymmetrical curved process. Ductus bursae tubular, posterior 1/3 well-sclerotised and twisted, remainder membranous. Cervix bursae elongated, bearing the ductus seminalis, with a weakly sclerotised bulla. Corpus bursae ovate, weakly sclerotised; signum absent.

#### Diagnosis

*Eulocastra sasakii* can be distinguished from other East Asian congeners by the following combination of adult and genitalic characters: fore-wing pattern with a distinct fuscous basal area, a well-defined medial band and three separated subterminal patches; hind-wing uniformly creamy-ivory; male genitalia with asymmetrical valvae bearing a strongly developed ampullar process; and female genitalia with a distinctly twisted ductus bursae and the absence of a signum.

It differs from *E. shibaharai* Kishida, 2010 (Kishida 2010: 61) not only in the fore-wing pattern (presence of a distinct medial band and non-uniformly suffused distal half), but also in the male genitalia, particularly the asymmetrical valva and strongly developed ampullar process (vs. valva symmetrical and processes weakly developed).

From *E. excisa* (Swinhoe, 1885) (Kishida 2011: 206), it differs in the fore-wing pattern, particularly in the presence of a more distinctly defined medial band with a relatively straight inner margin (vs. medial band indistinct or irregular), the subterminal markings separated into distinct patches (vs. forming a broad, continuous dark area) and in the uniformly pale hind-wing (vs. with darker suffusion).

From *E. neoexcisa* Berio, 1954 (Butler 1889: 61; De Prins and De Prins 2026), it differs in having a distinct, undulate postmedial line (vs. faint and irregular), a distinct and continuous subterminal line (vs. interrupted) and, in the male genitalia, a strongly sclerotised distal portion of the valva (vs. weakly sclerotised), a large, triangular ampullar process (vs. relatively small and axe-shaped), an elongate triangular harpe with a blunt apex (vs. broad and flattened, gradually tapering distally) and an aedeagus with a small process on the carina (vs. absent).

#### Distribution

Cambodia (Seima), Japan (Aichi, Akita, Fukui, Gifu, Mie) (Sugi 1985, Gifu Prefecture 2010, Kishida 2011, Fukui Prefecture 2016, Aichi Environmental Research Center 2020, Akita Prefecture Department of Living Environment. Nature Conservation Division 2020, Ministry of the Environment, Japan 2020, Mie Prefecture 2025).

#### Taxon discussion

Genitalic comparisons with some congeners remain limited, as the female genitalia of *E. shibaharai* have not been described and the genitalia of *E. excisa* are insufficiently documented.

The ostium bursae and ductus bursae of *E. sasakii* are naturally twisted, but were partially flattened during slide preparation to clarify their structure.

#### Host plants

*Molinia japonica* (Nakamura et al. 1996, Gifu Prefecture 2010, Aichi Environmental Research Center 2020, Mie Prefecture 2025).

## Discussion

The present study documents the first record of *Eulocastra sasakii* from Southeast Asia, based on specimens collected in Cambodia. Prior to this study, this species was regarded as endemic to Japan and listed as endangered in several regions, including Central (Aichi, Gifu and Mie Prefecture) and Northern Honshu (Akita Prefecture), where it is rarely observed or collected.

Until now, *Molinia japonica* Hack. has been reported as its only host plant, with a distribution restricted to temperate regions of East Asia, including Korea, Japan, Eastern Siberia and parts of eastern China. However, this species is not known to occur in the Indochina Peninsula, including Cambodia (Efloras 2023, POWO 2026).

The occurrence of *E. sasakii* in Cambodia, a region well outside the known distribution range of *M. japonica*, raises questions regarding its host plant associations. This finding suggests that the species may utilise alternative host plants in Southeast Asia, although this hypothesis remains to be tested. In Japan, the host plant has been referred to as *Moliniopsis japonica* by the Ministry of the Environment, Japan (Ministry of the Environment, Japan 2025); however, the author follows the usage of *Molinia japonica* in this study (Son et al. 2025).

This finding represents the first confirmed record of *E. sasakii* outside Japan, suggesting that the species may occur in both the Palaearctic and Oriental Regions. Although the examined specimens are consistent with *E. sasakii*, based on genital morphology, additional material and molecular data are required to confirm whether the Cambodian population is conspecific with the Japanese population.

In addition, the female genitalia of *E. sasakii* are described and illustrated for the first time, providing diagnostic characters that will facilitate future identification and comparative studies within the genus *Eulocastra*. Given that the present study is based on a limited number of specimens, further sampling across both the Oriental and eastern Palaearctic Regions will be essential to clarify the distribution, host plant associations and intraspecific variation of this species.

Furthermore, although *Eulocastra sasakii* shows a close external similarity to *Eulocastra quintana* (Swinhoe, 1885) described from India, as pointed out by Sugi (1990), a detailed morphological comparison, particularly of the genitalia, remains unfeasible at present. This is due to the unavailability of the type specimen and the absence of any subsequent records or detailed morphological data for *E. quintana* beyond the original description. Future discovery and examination of fresh material or type holdings of *E. quintana* will be essential to definitively clarify the specific boundaries and phylogenetic relationships between these allied taxa.

## Supplementary Material

XML Treatment for Eulocastra sasakii

## Figures and Tables

**Figure 1a.**
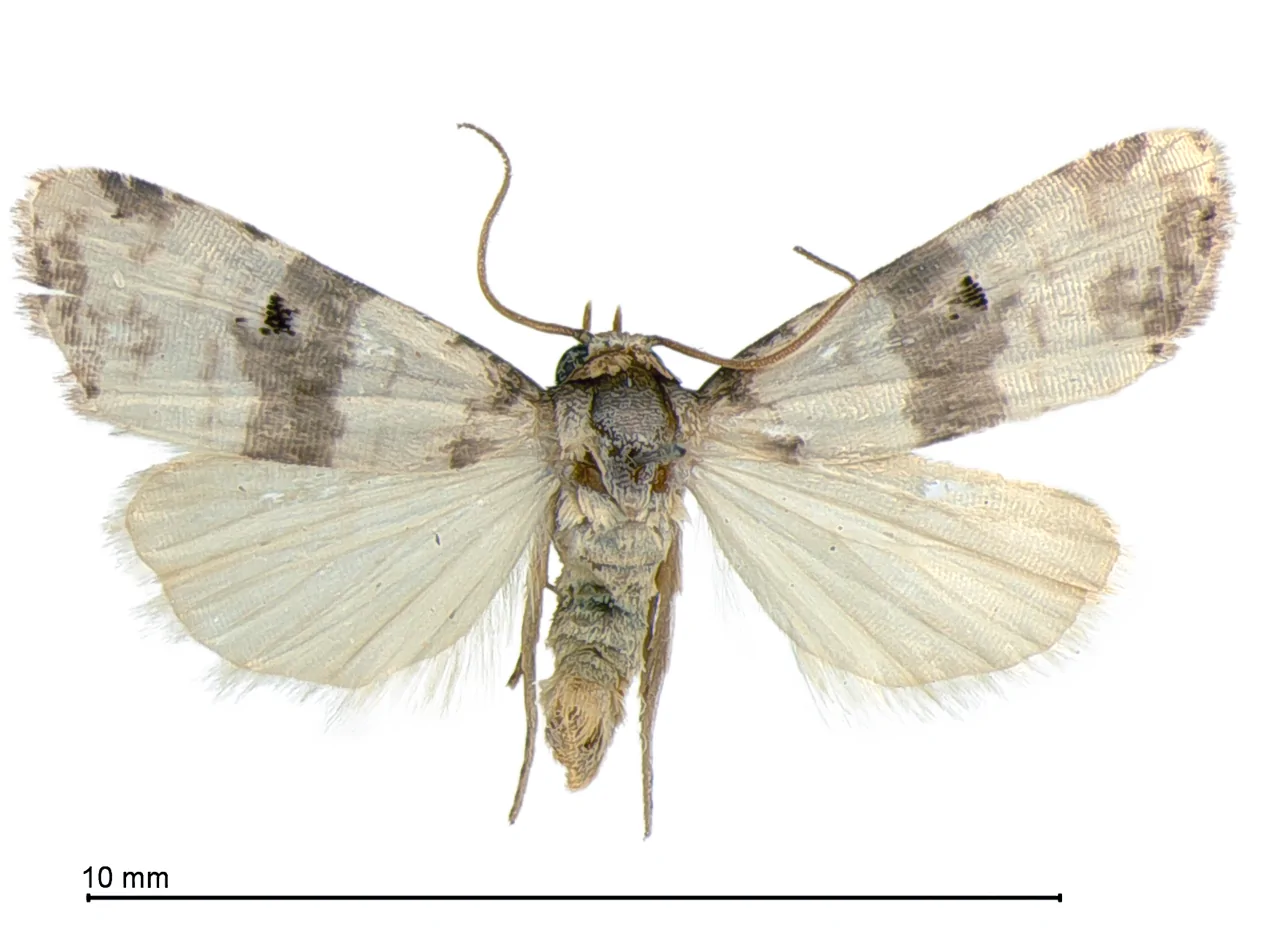
INU-12214, male;

**Figure 1b.**
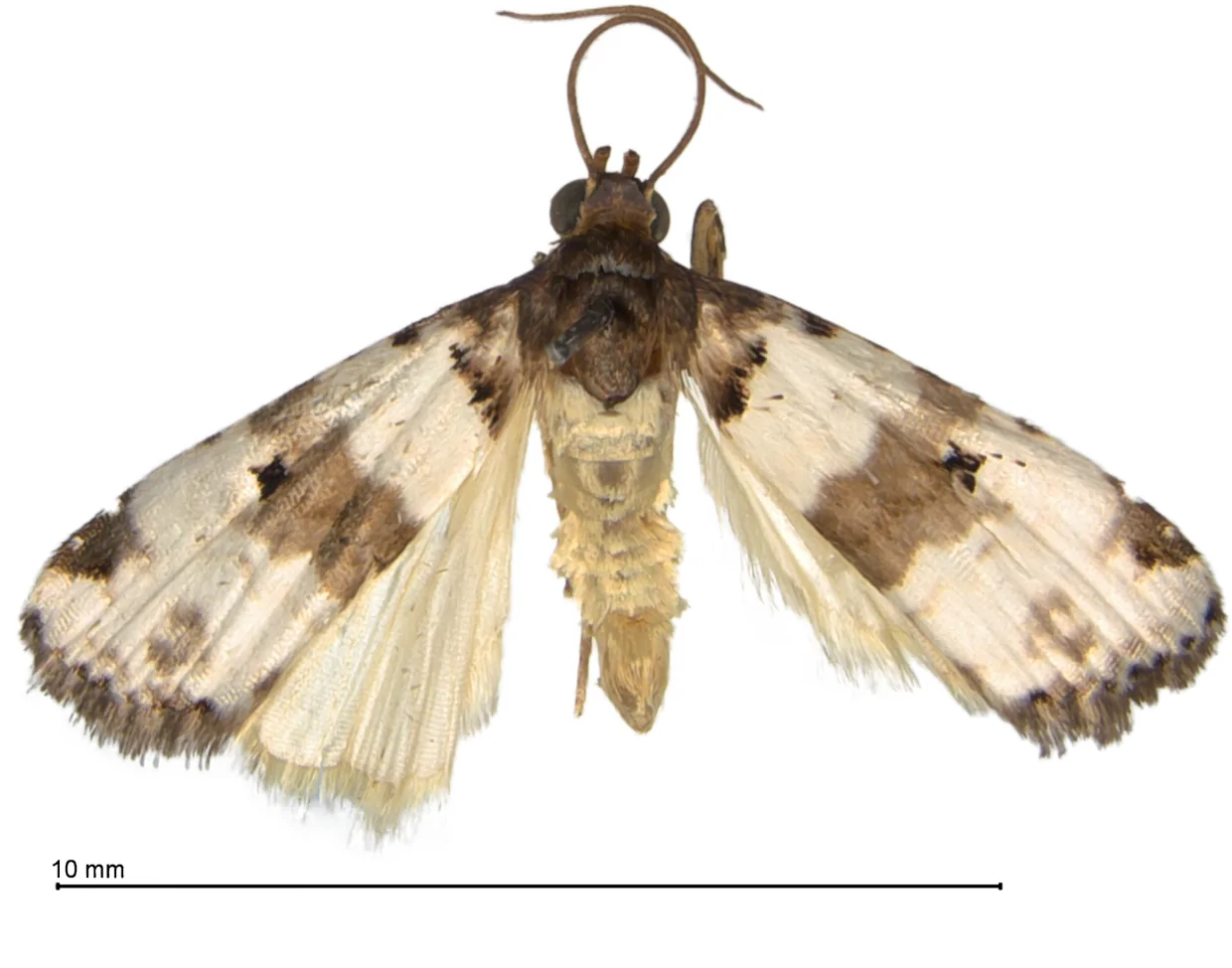
CYBG-0245, male;

**Figure 1c.**
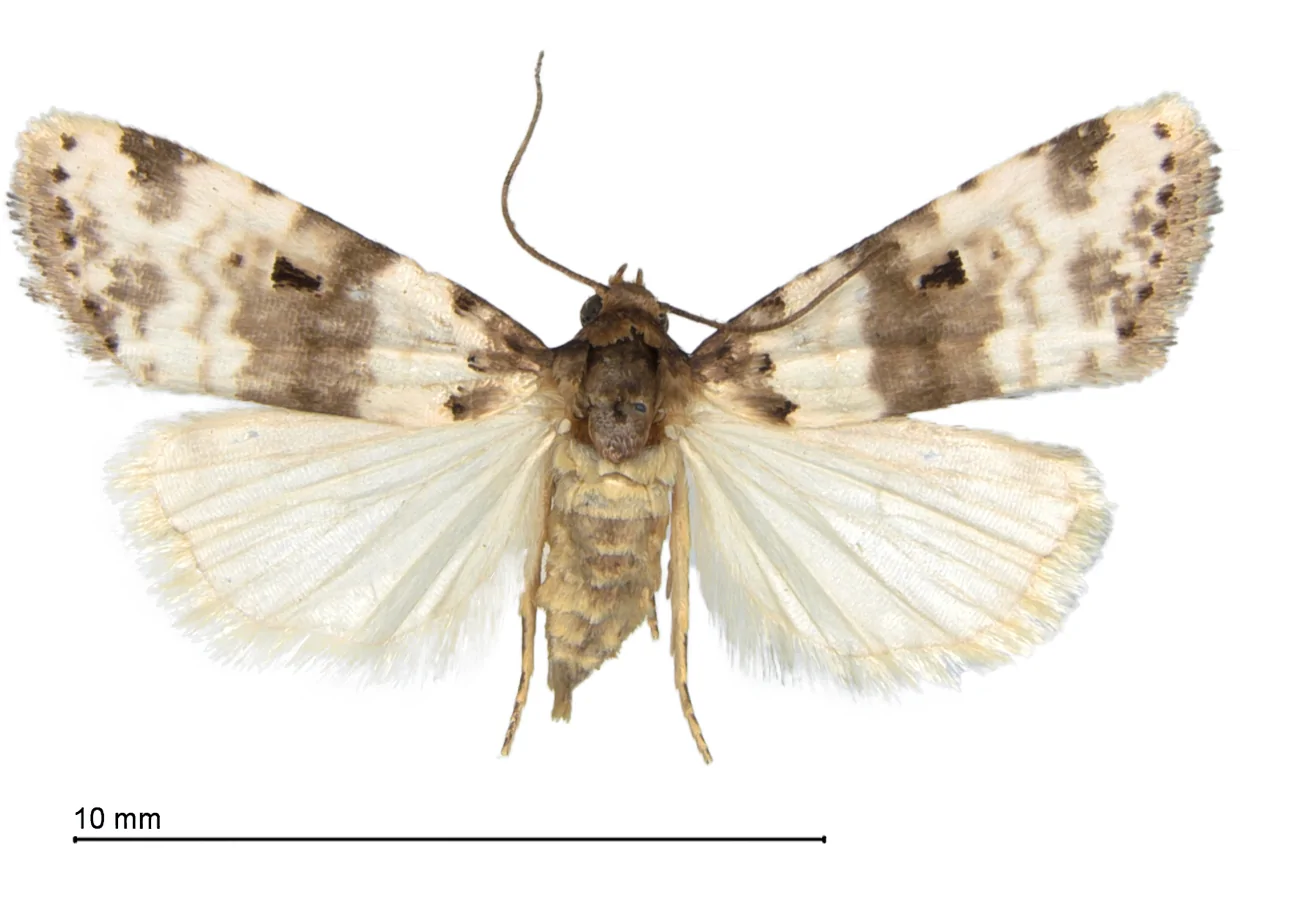
CYBG-0244, female;

**Figure 1d.**
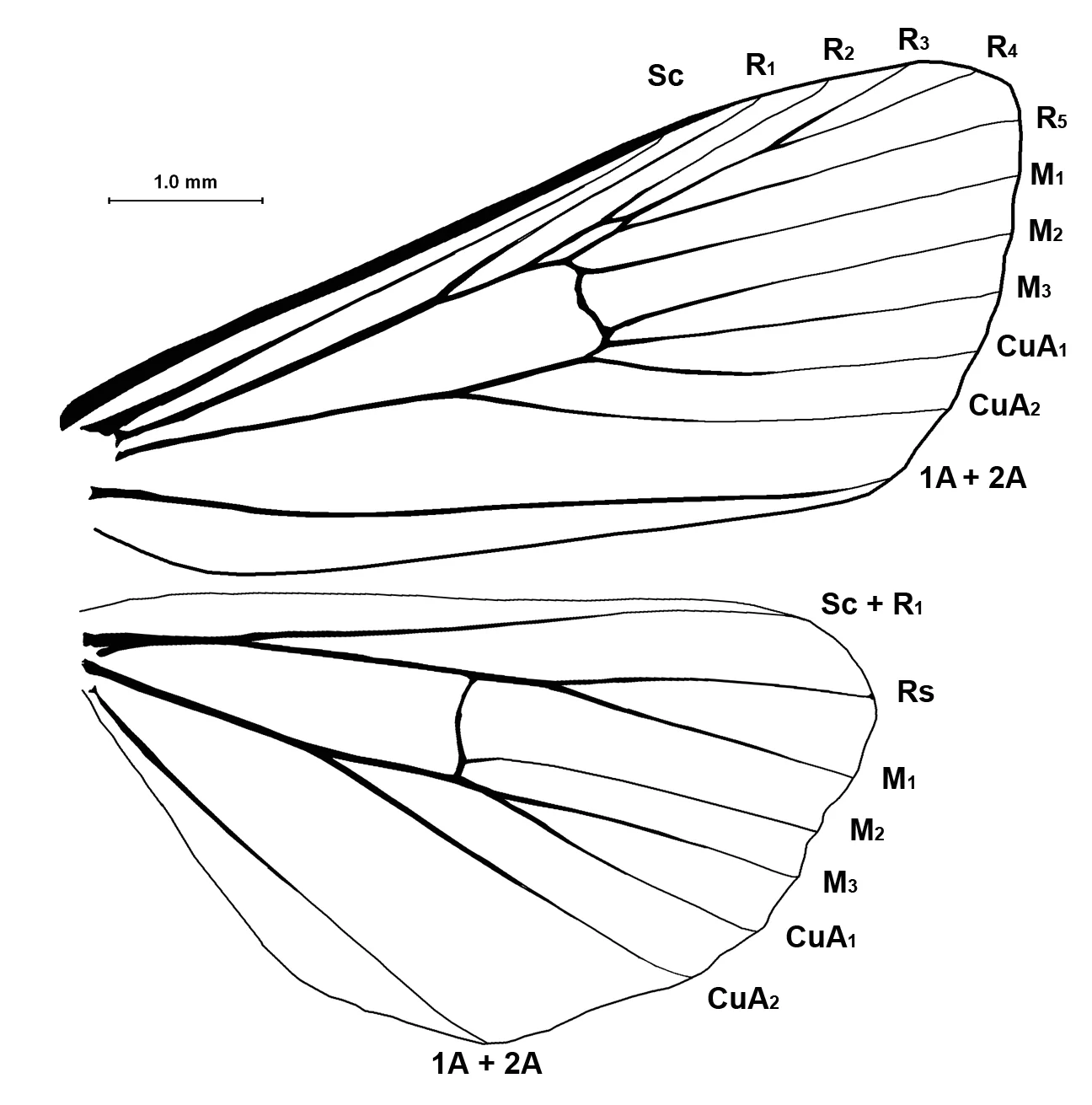
CYBG-0245V, wing venation of CYBG-0245.

**Figure 2a.**
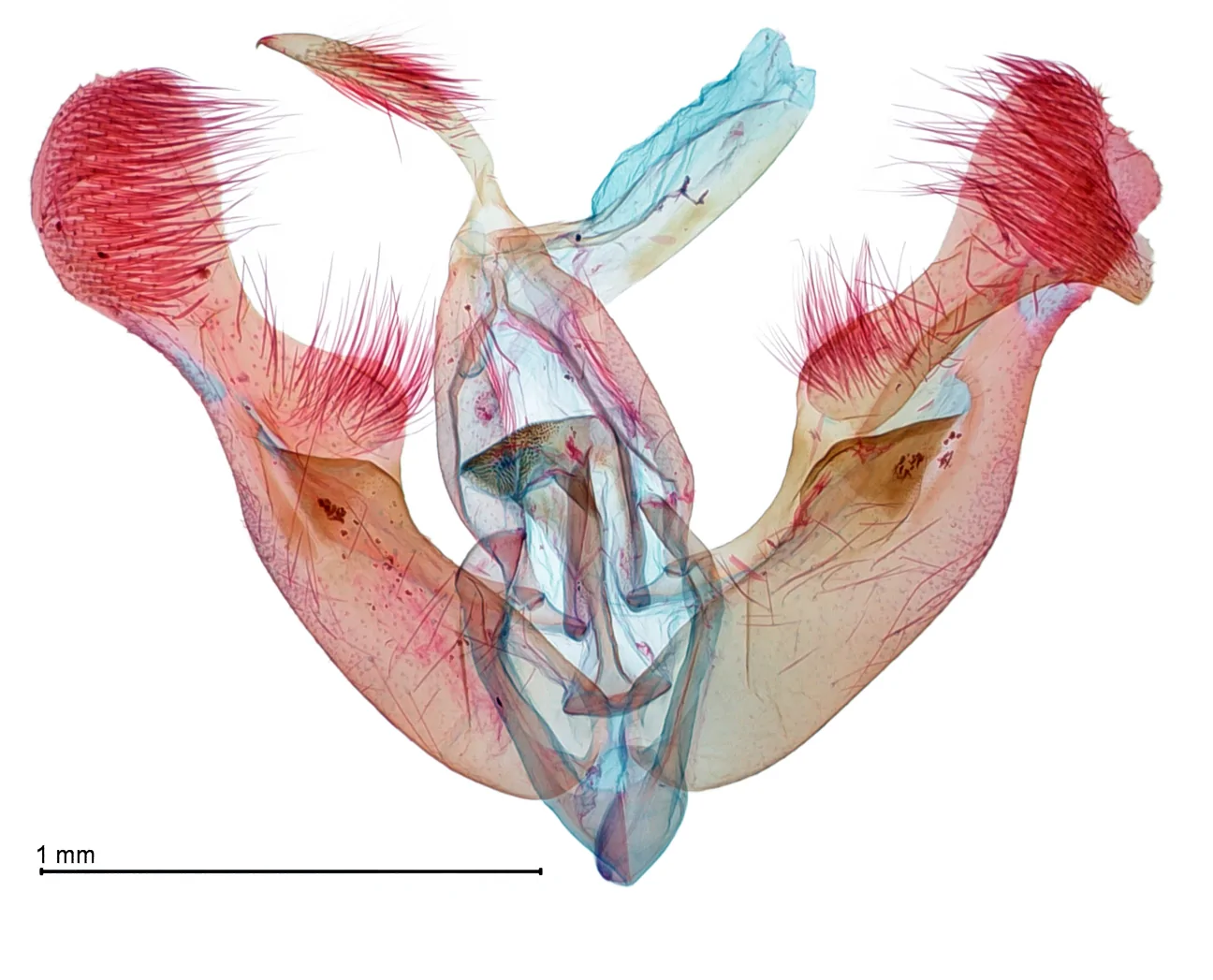
INU-12214, genital capsule;

**Figure 2b.**
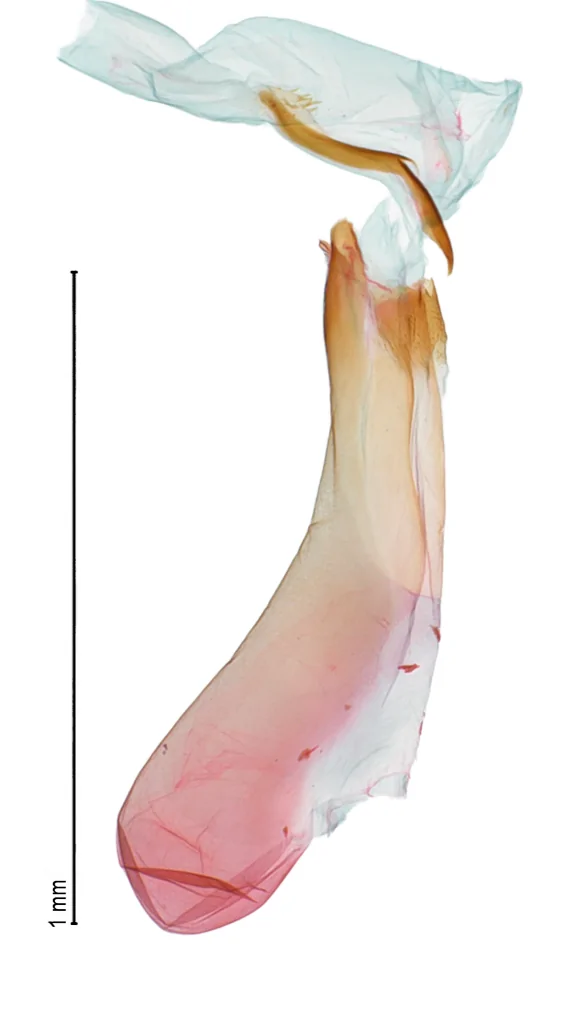
*ditto*, aedeagus;

**Figure 2c.**
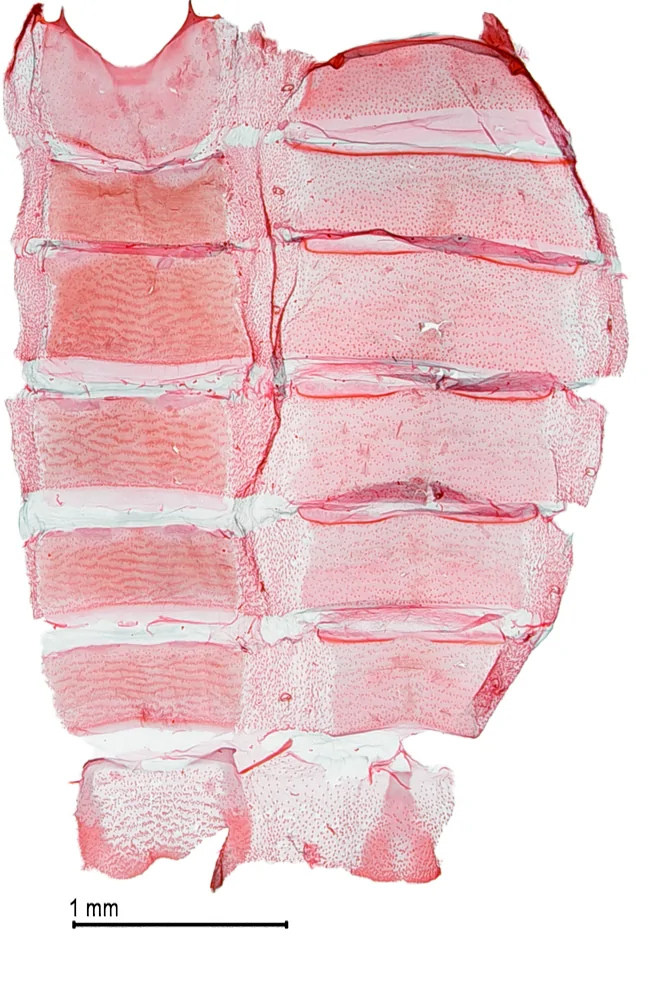
*ditto*, abdomen;

**Figure 2d.**
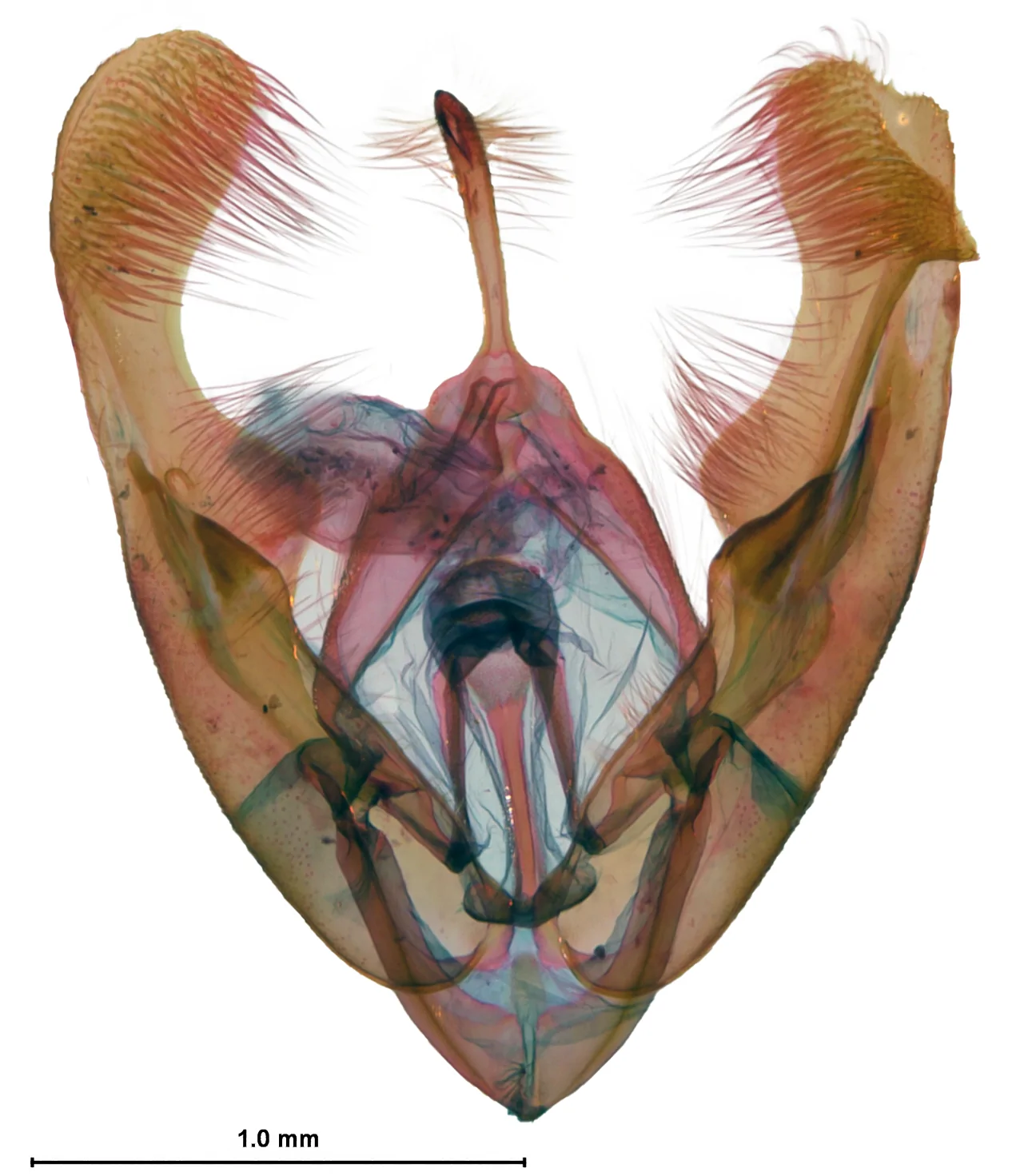
CYBG-0245, genital capsule;

**Figure 2e.**
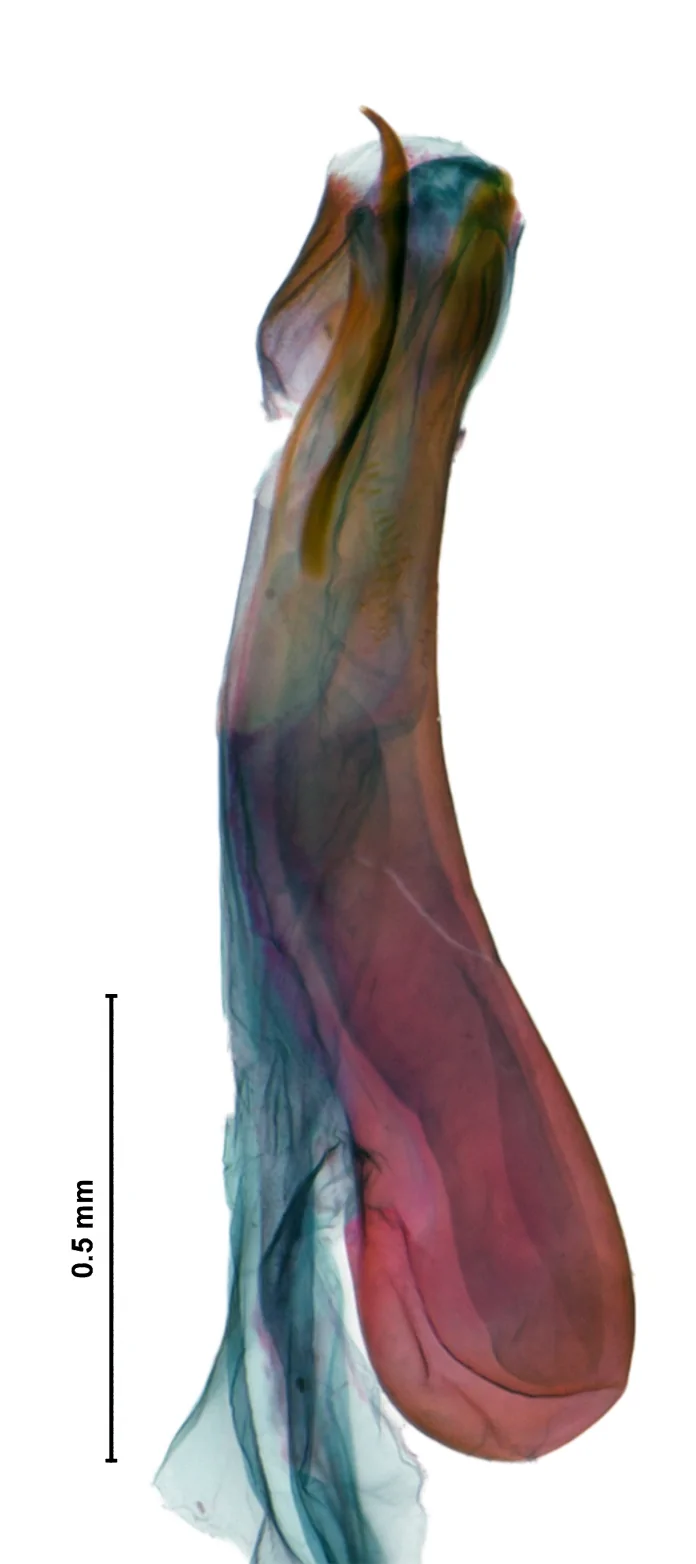
*ditto*, aedeagus;

**Figure 2f.**
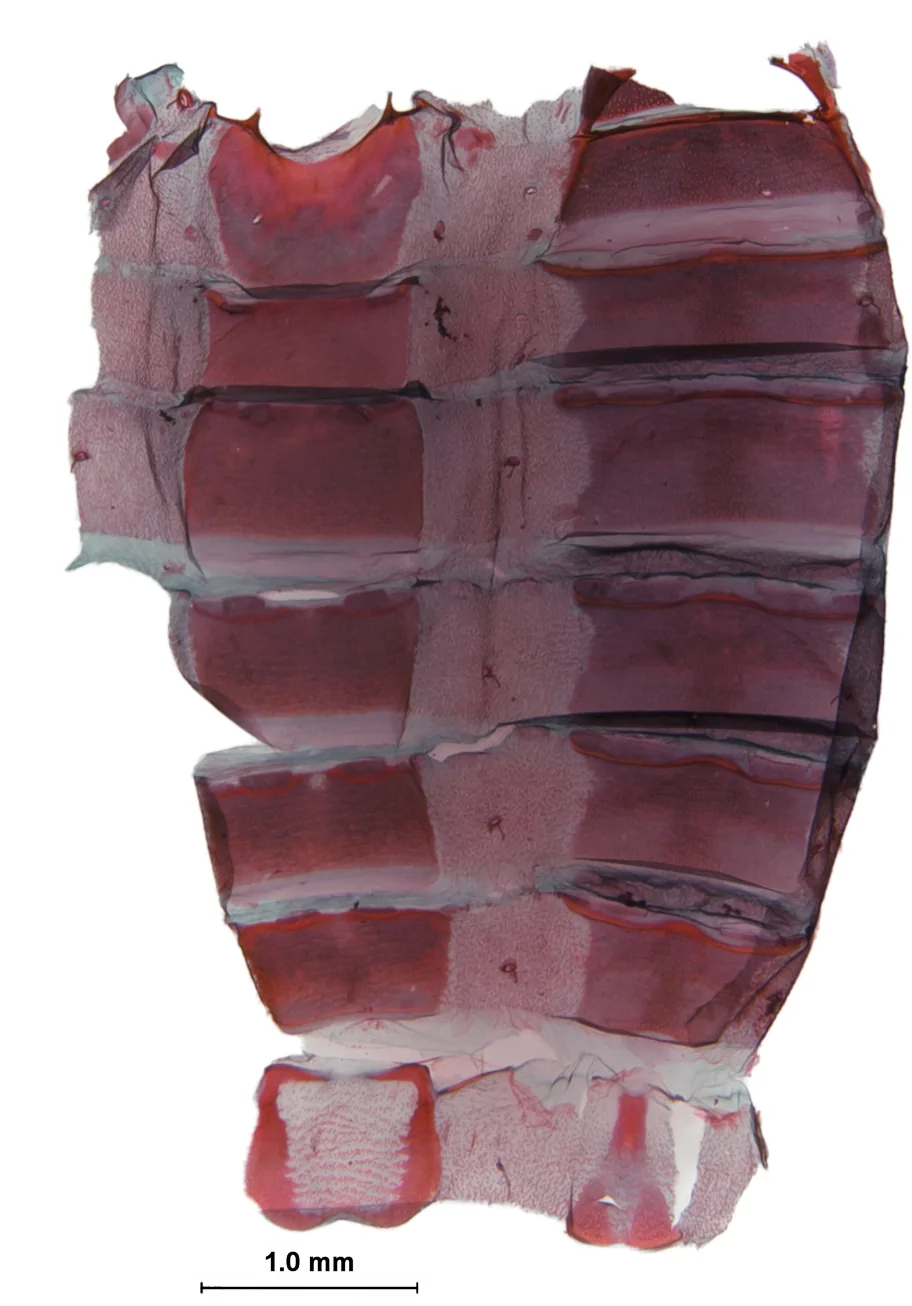
*ditto*, abdomen.
